# Increasing Access to Surgical Services in Sub-Saharan Africa: Priorities for National and International Agencies Recommended by the Bellagio Essential Surgery Group

**DOI:** 10.1371/journal.pmed.1000200

**Published:** 2009-12-22

**Authors:** Sam Luboga, Sarah B. Macfarlane, Johan von Schreeb, Margaret E. Kruk, Meena N. Cherian, Staffan Bergström, Paul B. M. Bossyns, Ernest Denerville, Delanyo Dovlo, Moses Galukande, Renee Y. Hsia, Sudha P. Jayaraman, Lindsey A. Lubbock, Charles Mock, Doruk Ozgediz, Patrick Sekimpi, Andreas Wladis, Ahmed Zakariah, Naméoua Babadi Dade, Peter Donkor, Jane Kabutu Gatumbu, Patrick Hoekman, Carel B. IJsselmuiden, Dean T. Jamison, Nasreen Jessani, Peter Jiskoot, Ignatius Kakande, Jacqueline R. Mabweijano, Naboth Mbembati, Colin McCord, Cephas Mijumbi, Helder de Miranda, Charles A. Mkony, Pascoal Mocumbi, Jean Bosco Ndihokubwayo, Pierre Ngueumachi, Gebreamlak Ogbaselassie, Evariste Lodi Okitombahe, Cheikh Tidiane Toure, Fernando Vaz, Charlotte M. Zikusooka, Haile T. Debas, for the Bellagio Essential Surgery Group (BESG)

**Affiliations:** 1 Makerere University, Kampala, Uganda; 2 University of California, San Francisco, San Francisco, California, United States of America; 3 Karolinska Institutet, Stockholm, Sweden; 4 University of Michigan, Ann Arbor, Michigan, United States of America; 5 World Health Organization, Geneva, Switzerland; 6 Belgian Technical Cooperation, Brussels, Belgium; 7 Institute of Tropical Medicine of Antwerp, Antwerp, Belgium; 8 University of Toronto, Toronto, Canada; 9 National Ambulance Services, Accra, Ghana; 10 Centre Hospitalier Régional, Dosso, Niger; 11 Kwame Nkrumah University of Science and Technology, Kumasi, Ghana; 12 Kenyan Society of Anesthesia, Nairobi, Kenya; 13 Belgian Technical Cooperation, Niamey, Niger; 14 Council on Health Research for Development (COHRED), Geneva, Switzerland; 15 University of Washington, Seattle, Washington, United States of America; 16 International Development Research Centre, Nairobi, Kenya; 17 Clinical Officer Training, Limbe, Malawi; 18 National University of Rwanda, Butare, Rwanda; 19 Mulago National Referral Hospital, Kampala, Uganda; 20 Muhimbili University of Health and Allied Sciences, Dar es Salaam, Tanzania; 21 Columbia University, New York, New York, United States of America; 22 Catholic University, School of Medicine, Beira, Mozambique; 23 World Health Organization, Brazzaville, Republic of Congo; 24 Medical School of Universite des Montagnes, Douala, Cameroon; 25 Ministry of Health and United Nations Population Fund, Asmara, Eritrea; 26 Belgium Technical Cooperation, Dakar, Senegal; 27 Université Cheickh Anta Diop, Dakar, Senegal; 28 Higher Institute of Health Sciences, Maputo, Mozambique; 29 HealthNet, Kampala, Uganda

## Abstract

In this Policy Forum, the Bellagio Essential Surgery Group, which was formed to advocate for increased access to surgery in Africa, recommends four priority areas for national and international agencies to target in order to address the surgical burden of disease in sub-Saharan Africa.

## Introduction

In sub-Saharan Africa, only 46% of births are attended by skilled personnel, compared to 96% in Europe (according to data for the African Region of the World Health Organization [WHO] from 2000 to 2008 [1]). In 2005, slightly over one quarter of a million women died from complications of childbirth [1]; most of these deaths could have been avoided by providing women with access to basic obstetric care and obstetric surgical care. On average, across sub-Saharan Africa, a population of 10,000 is served by two doctors and 11 nursing and midwifery personnel, compared to 32 and 79 respectively serving the same number of people in Europe (WHO data 2000–2007 [1]). A child born in sub-Saharan Africa in 2007 could expect to live only 52 years, which is 22 years less than its European counterpart [1]. Such starkly contrasting figures drive national and international efforts to build health system capacity to save lives and increase life expectancies in Africa. We argue that these efforts should include surgical capacity, a neglected component of a functioning health system.

The overall disease burden associated with surgical conditions in sub-Saharan Africa is estimated at 38 DALYS (disability adjusted life years) lost per 1,000 population. This estimate is higher than in other regions of the world, and is mainly due to injuries (15/1,000), obstetric complications (6/1,000), malignancies (3/1,000), perinatal conditions (3/1,000), congenital anomalies (3/1,000), and cataracts and glaucoma (2/1,000) [2]. The estimated cost per surgical DALY gained at a district hospital is in the range of US$19–102 [2]. By comparison, the basic immunization program in Africa costs under US$10/DALY averted, malaria prevention and treatment costs US$2–24/DALY averted, and oral rehydration therapy for diarrheal disease can cost around US$1,062/DALY averted [3]. Antiretroviral therapy for HIV infection in sub-Saharan Africa is estimated to be in the range of US$350–1,494/DALY averted [3]. Yet, the global health community has largely neglected surgical diseases when supporting health interventions in sub-Saharan Africa [4],[5].

The Bellagio Essential Surgery Group (BESG)—a network of surgeons, anesthesiologists, public health professionals, economists, and policy makers—was formed to advocate for increased access to surgery in Africa to reduce the surgical burden of disease (Box 1). The BESG builds on and collaborates with the significant work of the WHO in promoting surgical and trauma care. The WHO Global Initiative for Emergency and Essential Surgical Care is a multidisciplinary group of stakeholders committed to reducing death and disability in injuries, pregnancy-related complications, and congenital anomalies, and was the first coordinated effort on emergency and essential surgical care [6]. WHO Essential Trauma Care Project aims to set reasonable, affordable minimum standards for the care of injured persons worldwide and defines the human and physical resources necessary to ensure these services [7].

**Box 1. Bellagio Essential Surgery Group (BESG)**

The BESG (http://www.essentialsurgery.org/bellagio/docs/2008_kampala_essential_surgery_report.pdf) is a multidisciplinary, international network focused on developing collaborative strategies to increase access to surgical services across sub-Saharan Africa. With support from the Rockefeller Foundation, the BESG first met in Bellagio, Italy in June 2007 and, with support from the Bill &Melinda Gates Foundation, the group met in Kampala, Uganda in July 2008 to develop specific cross-country strategies and implementation plans to address the large disease burden due to surgical conditions in sub-Saharan Africa. The BESG welcomes participation from all health professionals working to decrease disparities in access to health care and to reduce the burden of disease experienced by poor people living in sub-Saharan Africa.

We recommend four priority areas for national and international agencies to target in order to begin to address the surgical burden of disease in sub-Saharan Africa. The priority areas are based on the experience of projects in Ghana, Malawi, Mozambique, Niger, Tanzania, and Uganda and the WHO. The consensus statement from the 2008 BESG Kampala meeting can be found in Text S1. The context guiding our recommendations is the lack of even the most basic surgical services in rural areas and small towns throughout sub-Saharan Africa and our recognition of the broader role of surgery in strengthening health systems and fulfilling a basic human right to health care. Our definition of a surgical condition is one that “requires suture, incision, excision, manipulation, or other invasive procedure that usually, but not always, requires local, regional, or general anesthesia” as proposed by Debas et al. [2]. This definition includes major obstetric interventions.

## Recommendation 1: Strengthen Surgical Services at District Hospitals

Surgical care is usually concentrated in overloaded specialist referral hospitals that are inaccessible to patients who are unable or unwilling to travel. Those patients who do reach a health facility often arrive at a relatively advanced state of disease when the curative window may have passed. For example, 77% of patients with breast cancer evaluated in a tertiary Ugandan health facility presented in advanced stage compared with a much smaller fraction in high-income countries [8].

Debas et al. propose that a properly equipped district hospital in a low-income country could perform: emergency surgery for obstetric complications; abdominal emergencies and basic thoracic and head injuries; simple orthopedic care for extremity fractures, dislocations, and amputations; burn care; and uncomplicated general surgery for hernias, anorectal conditions, and treatment and control of surgical infections [2]. However, many district hospitals in sub-Saharan Africa lack the capacity to provide such care. In a survey of district health facilities in Kenya, Uganda, Southern Sudan, and Rwanda, Pearson and Shoo found that only 2%–18% of all expected direct obstetric complications were treated [9].

The obstacles to providing obstetric care recorded by Pearson and Shoo reflect the general shortcomings of district-level care, including: shortage of trained staff, poor basic infrastructure, inadequate supplies of drugs and essential equipment, poor working conditions, low staff morale, lack of communication and referral facilities, costs to patients of treatment, and poor management [9]. National strategies are required to upgrade such facilities and should simultaneously focus on the human and other resources needed to perform basic surgical procedures. A functioning surgical unit at a local district hospital that is truly multipurpose can respond to a wide variety of emergent and routine conditions and childbirth complications. The presence of such a unit would help to ensure a functioning blood bank, a clinical laboratory, and the emergency transport and communication systems of a hospital, and thus improve its overall effectiveness. The availability of such surgical capacity should also increase the confidence of communities that their health services can respond effectively to emergencies, and avert deaths or disabilities that can result in long-term absence from work or inability to earn a living.

## Recommendation 2: Improve Systems for the Delivery of Trauma Care

Injury, including road traffic injuries, accounts for nearly 10% of all DALYs lost in sub-Saharan Africa [2], and it is predicted that injury will contribute 20% of the global burden of disease by 2020 [10]. Experience in high-income countries has shown that improving the organization and planning for trauma care can consistently decrease deaths among all treated trauma patients by 15%–20%, and trauma systems have decreased 50% of medically preventable deaths [11]. Yet there is little support for African countries to improve their trauma care systems.

The components of a trauma care system that need to be addressed include: improvements in pre-hospital care, patient management, strengthening of care at clinics and hospitals, streamlining of the referral process which includes patient transportation between institutions, instituting financing mechanisms to remove financial barriers to care, and ensuring adequate data with which to monitor the quality of care provided. A growing number of countries are reporting improvements in one or more of these trauma system components (Box 2). Successful programs need to be scaled up into improved countrywide trauma systems. These efforts can be promoted by ensuring that a person or unit within the ministry of health has the appropriate background and training and is adequately empowered to promote trauma system improvements. Many of the specific actions that ministries of health need to implement have been well addressed by World Health Assembly Resolution 60.22 [12] on trauma and emergency care services, which we urge governments throughout Africa to institute.

**Box 2. Trauma Services in Ghana**

In Ghana, two initiatives have shown improvements in the delivery of trauma care. A pilot program to provide commercial drivers with basic first aid training led to a documented improvement in the provision of key skills such as airway management, bleeding control, splint application, triage, and scene management in the pre-hospital care setting [32],[33]. Likewise, over the past 12 years, Kwame Nkrumah University of Science and Technology has provided continuing education to over 100 doctors from rural hospitals in order to strengthen trauma care at the district level. This week-long course has demonstrated improved skills in trauma care, including basic and advanced airway management, chest tube insertion, and management of open fractures [34]. Both of these initiatives are locally based, sustainable, low-cost, and eminently applicable in other African countries.

## Recommendation 3: Expand the Supply and Quality of Health Workers with Surgical Skills

There are not enough health workers trained to provide adequate surgical services in specialist or district hospitals. This situation reflects both the critical shortage of health workers throughout sub-Saharan Africa [13],[14] and a lack of surgical specialists. Although some surgical procedures require highly skilled staff, a specialist surgeon is not required to perform many of the procedures described by Debas et al. [2]. An audit of eight district hospitals in Zambia, for example, concluded that nonsurgeons could have been trained to provide 86% of all operations performed [15]. The reality, in any case, is that the operations are not being performed by surgeons; in Uganda, Ozgediz recorded 3,621 operations (53% obstetric) at four district hospitals in a year, but noted that there was only one obstetric surgeon in one of the hospitals and no surgeons at the other three [16].

Some countries, such as Senegal, prefer to generate sufficient specialist surgeons to operate on patients in well-equipped tertiary referral hospitals as well as district hospitals. Other countries are training general physicians to perform basic surgery, for example in Niger (Box 3), and some, like Malawi (Box 4), train nonphysicians to perform specific surgical procedures. It is essential, of course, that nonspecialist health workers are properly supervised and that their training programs are carefully evaluated. We suggest that there is sufficient experience with training nonsurgeons to establish mechanisms for accreditation and coordination of the training programs within and across countries, and to conduct objective evaluations of their outcomes.

**Box 3. National Strategies to Strengthen Surgical Care at the District Level: An Example from Niger**

In Niger, only 10% of women in need of emergency cesarean sections receive appropriate care, mainly because of distance to adequate facilities in specialist hospitals and lack of available transport [35]. Emergency surgical trauma care is similarly inaccessible. Even in rare cases when emergency transport is available in Niger, over 50% of patients—mostly with obstetric and surgical conditions—refuse emergency evacuations, sometimes because they cannot afford the cost of transportation [36], and perhaps also because they do not want to move away from their families and communities.

In February 2007, the Government of Niger began to deploy teams of two general practitioners certified as having “capacity in district surgery” and to provide them with financial and other incentives to stay in the districts. These physicians, some of whom are already working at the district level, are trained for three months at the University of Niger's Faculty of Health Sciences and selected hospitals in Niamey, and for nine months in regional hospitals throughout the country. The Government also supports the training of specialists in general surgery, trauma, and obstetrics at the National Hospital and then assigns them to regional hospitals where they provide supervision and referral services for the district surgical teams [37].

**Box 4. Training Midlevel Health Professionals to Perform Surgery: An Example from Malawi**

In Malawi, as of 2003, there were only 15 trained surgeons of any specialty, including expatriates, to serve a population of 12 million and none of these were stationed at any of the district hospitals [38].

To address this acute surgical workforce shortage, in 2005 Malawi started piloting on-the-job training in surgery for nonphysician Clinical Officers. Although there was substantial enthusiasm for this pilot program, its effectiveness was undermined by the lack of a long-term career path or any additional salary support for the Clinical Officers who were trained. A bachelor's program is now being developed to address these issues. The highest achieving Clinical Officers who complete two years of on-the-job training at the district level are now given the opportunity to continue to a two-year advanced course on surgery, gynecology, and orthopedics/trauma under supervision at the Central Hospital where they learn how to deal with the most acute conditions and complicated cases [39].

The shortage in trained anesthesia providers and perioperative nursing care is also extreme in sub-Saharan Africa. Studies show that significant perioperative morbidity and mortality in these settings is preventable and that there are severe limitations to the delivery of safe anesthesia [17],[18]. The workforce skills (including choice of anesthesia) and infrastructure to ensure safe perioperative care must be built to meet anesthesia service needs in district hospitals and should be addressed in the comprehensive district health care package.

## Recommendation 4: Build Evidence to Inform Interventions to Improve Access to Surgery in Sub-Saharan Africa

There is little evidence to answer basic questions about the prevalence and incidence of surgical conditions and the provision of surgical interventions in sub-Saharan Africa. For example, the global burden of surgical disease as estimated by Debas et al. is, at best, a rough estimate. Weiser et al., in calculating the global volume of surgery, found data on surgical volume for only 20% of African countries compared to 54% of European countries [19].

The lack of information about surgery in sub-Saharan Africa was noted almost 20 years ago by Nordberg [20]. (Nordberg's research priorities along with those of the BESG and others are summarized in Box 5.) That Nordberg's research agenda remains unmet today may be partly because surgeons are not usually trained in the skills of policy, advocacy, or research and are ill-equipped to effectively argue for a broader role for surgical services.

**Box 5. A Research Agenda to Build the Evidence to Inform Policy-makers**

Building on the priorities listed by Nordberg [20] and others, the BESG recommends quantification of the:

- Burden of surgical disease, particularly at the country level [2],[40].
- Need for, access to, and outcomes of surgical care [20]; and of disparities in these indicators between socioeconomic groups and populations living in urban and rural areas.
- Iatrogenic expenditure related to surgical care. Since the economic consequences of having a major operation may be substantial, health financing strategies that minimize this economic burden need to be developed [41],[42].
- Availability and use of surgery-related resources such as human resources, equipment, and supplies [20].
- Coverage, quality, and effectiveness of interventions to strengthen the delivery of essential surgical services [43].

## Moving the Agenda Forward

The BESG was formed in order to stimulate national and international efforts to improve access to surgical services in sub-Saharan Africa. Our recommendations are consistent with other recent calls to include surgery in the global health agenda [5],[16],[21],[22]. We are not proposing a new vertical initiative; rather, we advocate for the integration of surgical care into ongoing programs. Surgical services can be mainstreamed into health systems through ongoing international and national initiatives.

The current revival of primary health care [23] provides an opportunity to properly integrate surgical services at the district level as part of an essential health services package. Obstetricians have already led the way by advocating for and providing surgical services at the district level to handle obstetric complications [24]. Such work may be complemented and enhanced by ensuring that the surgical services provided are comprehensive enough to serve the population's other acute and chronic surgical needs [25]. This is in line with other calls to integrate programs across health systems [26]–[29]. We hypothesize that investment in surgical services will strengthen not only hospital services but the health system itself. District demonstration projects need to be designed and funded to provide the evidence to test this hypothesis.

National governments need to commission country assessments to determine realistic and innovative solutions to reduce surgical mortality and morbidity; consider expanding the surgical competency base to other health care personnel and implement effective strategies to attract and retain skilled health workers, especially at the district level; and invest in improvements and expansion of infrastructure and equipment to strengthen surgical and trauma care. Many private not-for-profit and international nongovernmental organizations provide a large volume of general and specialty surgical services in Africa [30],[31]. Often, these organizations are the sole providers of services, building local capacity in austere environments. Collaboration of governments with these organizations will be critical to moving our recommendations forward.

We call on surgeons, public health researchers, health economists, epidemiologists, and social scientists to collaborate to determine research priorities, institute training in appropriate research methods, encourage funders to support surgical research projects and undertake such work together. Lastly, we also call on surgeons, through their regional and national professional associations, to look beyond the walls of their operating theatres to involve themselves in advocacy, training, research, and health service management.

## Supporting Information

Text S1 The crisis in surgical services in Africa.
(0.35 MB DOC)

## Abbreviations

DALYs: disability adjusted life years
BESG: Bellagio Essential Surgery Group
WHO: World Health Organization
COHRED: Council on Health Research for Development
