# Supplementary material for: Increasing Access to Surgical Services in Sub-Saharan Africa: Priorities for National and International Agencies Recommended by the Bellagio Essential Surgery Group
Source: PLoS Med. 2009 Dec 22;6(12):e1000200. doi: 10.1371/journal.pmed.1000200 (PMC2791210; doi:10.1371/journal.pmed.1000200)
Supplement: Text S1 — The crisis in surgical services in Africa. (0.35 MB DOC) [file pmed.1000200.s001.doc]

Text SI
